# Protective Effects of Dioscin Against Doxorubicin-Induced Hepatotoxicity *Via* Regulation of Sirt1/FOXO1/NF-κb Signal

**DOI:** 10.3389/fphar.2019.01030

**Published:** 2019-09-13

**Authors:** Shasha Song, Liang Chu, Huifang Liang, Jin Chen, Junnan Liang, Zhao Huang, Bixiang Zhang, Xiaoping Chen

**Affiliations:** Hepatic Surgery Center, Tongji Hospital, Tongji Medical College, Huazhong University of Science and Technology, Clinical Medicine Research Center for Hepatic Surgery of Hubei Province, Key Laboratory of Organ Transplantation, Ministry of Education and Ministry of Public Health, Wuhan, China

**Keywords:** dioscin, doxorubicin, liver injury, Sirt1/FOXO1/NF-κB signal, oxidative stress, inflammation, apoptosis

## Abstract

Doxorubicin (Dox), an antitumor antibiotic, has therapeutic effects on many kinds of tumors. However, Dox can produce some serious side effects that limit its clinical application. Thus, exploration of effective drug targets or active lead compounds against Dox-induced organ damage is necessary. Dioscin, one natural product, has potent effects against Dox-induced renal injury and cardiotoxicity. However, the effects of dioscin on Dox-induced hepatotoxicity have not been reported. In this study, the results showed that dioscin significantly ameliorated Dox-induced cell injury, reduced reactive oxygen species (ROS) level, and suppressed cell apoptosis in alpha mouse liver 12 (AML-12) cells caused by Dox. *In vivo*, dioscin evidently decreased the levels of alanine transaminase (ALT), aspartate transaminase (AST), malondialdehyde (MDA); increased the levels of superoxide dismutase (SOD), glutathione (GSH), and glutathione peroxidase (GSH-Px); and alleviated liver injury. Mechanism study showed that dioscin remarkably up-regulated the expression levels of silent information regulator 1 (Sirt1) and heme oxygenase-1 (HO-1) *via* increase of the nuclear translocation of NF-E2-related factor 2 (Nrf2) and suppressed the expression levels of forkhead box protein O1 (FOXO1) and kelch-like ECH-associated protein-1 (Keap1) to inhibit oxidative stress. Furthermore, dioscin obviously decreased the nuclear translocation of nuclear factor κB (NF-κB) and the mRNA levels of tumor necrosis factor alpha (TNF-α), interleukin 1β (IL-1β), and interleukin 6 (IL-6) to suppress inflammation. Meanwhile, dioscin significantly regulated tumor suppressor P53 (P53) expression level and BCL-2-associated X (BAX)/BCL-2 apoptosis regulator (BCL-2) ratio to inhibit cell apoptosis. These results were further validated by knockdown of Sirt1 using siRNA silencing in AML-12 cells, which confirmed that the target of dioscin against Dox-induced hepatotoxicity was Sirt1/FOXO1/NF-κB signal. In short, our findings showed that dioscin exhibited protective effects against Dox-induced liver damage *via* suppression of oxidative stress, inflammation, and apoptosis, which should be developed as one new candidate for the prevention of Dox-induced liver injury in the future.

## Introduction

Doxorubicin (Dox) is one of the most effective chemotherapeutic drugs for the treatment of lung, gastric, ovarian, breast, thyroid, sarcoma, and pediatric cancers (Cristina et al., 2009; Thorn et al., 2011; Dragojevic-Simic et al., 2013). However, long-term use of Dox can produce serious side effects on non-tumor tissues, and thus, its clinical application is limited (Reddy et al., 2012). There are numerous side effects including hepatotoxicity, nephrotoxicity, and cardiotoxicity, and the toxic effects on the reproductive system and nervous system produced by Dox cannot be reversed (Okunewick et al., 1985; Eugene et al., 2000; Saad et al., 2001; Mihailovic-Stanojevic et al., 2008; Dragojevic-Simic et al., 2013; Roomi et al., 2014; Pugazhendhi et al., 2018). In addition, the metabolites of Dox by hepatic microsomal enzymes and cytoplasmic reductase are adriamycin and hepatotoxic glycosides (Camaggi et al., 1988; Ganey et al., 1988). As the largest metabolic organ in the body, the liver plays critical roles in metabolism, which can also be easily damaged by Dox. Hence, exploration of active lead compounds against Dox-induced liver damage is necessary.

Drug-induced hepatotoxicity may occur through the formation of free radicals and the production of reactive oxygen species (ROS), which can cause oxidative damage to organs (Minotti et al., 2004; Qin et al., 2008; Sassocerri et al., 2010; Aryal et al., 2014). In addition, the activation of inflammatory response has been found in Dox-induced cardiotoxicity, which can be reduced by suppressing nuclear factor κB (NF-κB) (Ashikawa et al., 2004; Austin et al., 2016; Zhang et al., 2019). Some studies have confirmed that Dox-derived ROS can adjust tumor suppressor P53 (P53) signal, release cytochrome *c*, and activate caspase-3 to promote apoptosis (Mukherjee et al., 2004; Morsi et al., 2006; Paweorn et al., 2015). Therefore, simultaneous inhibition of oxidative stress, inflammation, and apoptosis should be one effective method to treat Dox-induced hepatotoxicity.

Silent information regulator 1 (Sirt1), a NAD^+^-dependent class III histone deacetylase, has the ability to deacetylate key metabolic players including peroxisome coactivator 1 alpha, which is also a master regulator of oxidative metabolism and one inducer of oxidative stress protection systems such as manganese superoxide dismutase (SOD) and catalase (Danz et al., 2009). Sirt1 can increase cell resistance and survival from stress to protect against Dox-induced oxidative damage and cell death. Overexpression of Sirt1 can protect the heart from oxidative stress through up-regulating the actions of antioxidants (Danz et al., 2009; Yang et al., 2015). Furthermore, previous studies have demonstrated that Sirt1 can suppress NF-κB and adjust P53 signal, culminating in regulating cardiomyocyte inflammation and apoptosis (Han et al., 2008; Yang et al., 2015). Hence, Sirt1 plays critical roles in regulating inflammation, oxidative stress, and apoptosis. However, to the best of our knowledge, there are no mechanistic studies on the roles of Sirt1 in Dox-induced hepatotoxicity.

**Figure 1:**
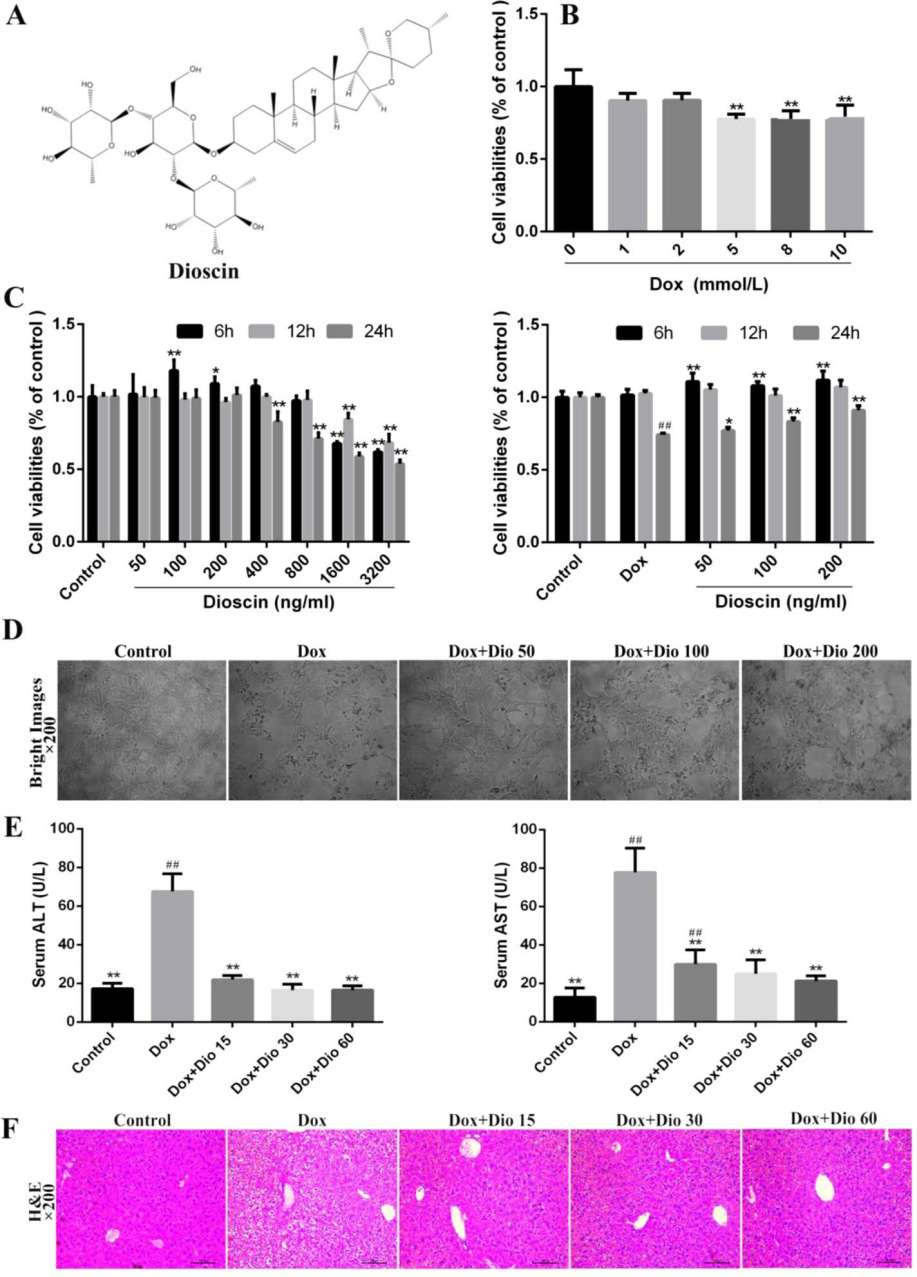
Dioscin inhibits Dox-induced AML-12 cell damage and liver injury in mice. **(A)** Chemical structure of dioscin. **(B)** Dox-induced nephrotoxicity on AML-12 cell. **(C)** Cytotoxicity of dioscin on AML-12 cells and the effects of dioscin on cell viability induced by Dox. **(D)** Effects of dioscin (50, 100, and 200 ng/ml) for 24-h pretreatment on the cellular morphology and structure of AML-12 cells by bright image (200× magnification) investigation. **(E)** Effects of dioscin on AST and ALT levels in mice. **(F)** H&E staining (200× original magnification) of the liver tissue in mice. All data are expressed as the mean ± SD (*n* = 5 for *in vitro* test and *n* = 8 for *in vivo* test). *p < 0.05, **p < 0.01 compared with the model group. ^##^ *p* < 0.01 compared with the control group. ALT, alanine transaminase; AML-12, alpha mouse liver 12; AST, aspartate transaminase; Dox, doxorubicin; H&E, hematoxylin and eosin.

With the development of modern pharmacology and molecular biology, the potential of natural products and Chinese herbal compounds for the prevention and treatment of human diseases has attracted more and more attention in recent years. Dioscin (shown in **Figure 1A**), a naturally derived triterpenoid saponin, has potent effects against various cancers (Qi Y. et al., 2016; Si et al., 2016; Zhao et al., 2016; Han et al., 2017; Tao et al., 2017; Xu L. et al., 2019) and protective activities against organ damage (Qi et al., 2015; Qi M. et al., 2016;Qiao et al., 2017; Zheng et al., 2017; Zhu et al., 2017; Han et al., 2018; Hu et al., 2018; Qi et al., 2019), which also has active actions against metabolic diseases including diabetes, hyperuricemia, obesity, and osteoporosis (Yin et al., 2015; Tao et al., 2016; Xu et al., 2017; Tao et al., 2018). Actually, beneficial therapeutic effects of dioscin on acute liver injury through alleviation of oxidative stress and suppression of inflammation have been studied, which can also promote liver regeneration (Gu et al., 2015; Liu et al., 2015; Xu et al., 2015; Zhang et al., 2015; Yao et al., 2016; Zhang et al., 2016; Qi et al., 2017). Also, dioscin shows protective effects against Dox-induced nephrotoxicity and cardiotoxicity (Injac et al., 2008; Qi et al., 2018). Based on these prospective findings, we speculated that dioscin might exert protective effects against Dox-induced hepatotoxicity, and we designed *in vivo* and *in vitro* experiments to verify our hypothesis.

## Materials and Methods

### Chemicals and Materials

Dioscin (purity > 98%) was obtained from *Dioscorea nipponica* Makino (Yin et al., 2010), which was dissolved in 0.5% carboxymethyl cellulose sodium (CMC-Na) for *in vivo* experiments and in 0.1% dimethyl sulfoxide (DMSO) for *in vitro* tests. The alanine transaminase (ALT), aspartate transaminase (AST), malondialdehyde (MDA), superoxide dismutase (SOD), glutathione (GSH), and glutathione peroxidase (GSH-Px) kits were from Nanjing Jiancheng Institute of Biotechnology (Nanjing, China). 3-(4,5-Dimethylthiazol-2-yl)-2,5-diphenyltetrazolium bromide (MTT) was provided by Roche Diagnostics (Basel, Switzerland). The bicinchoninic acid (BCA) protein assay kit, cell lysis buffer, and phenylmethanesulfonyl fluoride (PMSF) were obtained from Beyotime Institute of Biotechnology (Jiangsu, China). Dox and were purchased from Sigma-Aldrich (St. Louis, MO, USA).

### Cell Culture

Alpha mouse liver 12 (AML-12) cells (Shanghai Institute of Biochemistry and Cell Biology, China) were cultured in Dulbecco’s modified Eagle medium (DMEM) and Ham’s F12 medium with 5 μg/ml of insulin, 5 μg/ml of transferrin, 5 ng/ml of selenium, 40 ng/ml of dexamethasone, and 10% fetal bovine serum, which were maintained in a humidified atmosphere of 5% CO_2_ and 95% O_2_ at 37°C.

### Dox-Induced Cell Injury

Dox was prepared to make a series of working dilutions in serum-free DMEM. The AML-12 cells were plated in 96-well plates at a density of 8 × 10^4^ cells/ml per well for 24 h. Then, the medium was removed, and 100 μL of sample solution with various concentrations of Dox (0, 1, 2, 5, 8, and 10 mM) was added under different treatment times for 24 h. After 10 μL of MTT stock solution (5 mg/ml) was added, the plates were incubated for another 4 h at 37°C, and DMSO (100 ml/well) was added to dissolve formazan crystals. The absorbance was measured with a microplate reader (Thermo, USA) at 490 nm, the results were normalized to control unwanted sources of variation, and the cell morphology was imaged with a phase contrast microscope (Nikon, Japan). Then a suitable concentration of Dox on cell injury was optimized.

### Dioscin Toxicity Assay

AML-12 cells were plated in 96-well plates at a density of 8 × 10^4^ cells/ml for 24 h and treated with various concentrations of dioscin (0, 50, 100, 200 400, 800, 1,600, and 3,200 ng/ml) for 6, 12, and 24 h at 37°C, and the cells were then analyzed according to MTT method. The absorbance of the samples was quantified at 490 nm using a phase contrast microscope (Nikon, Japan).

### Cell Viability Assay

AML-12 cells were seeded in 96-well plates at a density of 8 × 10^4^ cells/ml for 24 h and then pretreated with various concentrations of dioscin (50, 100, or 200 ng/ml) for 6, 12, and 24 h before being challenged with Dox (5 μg/ml) for 24 h. The cells in the model group were cultured without dioscin pretreatment, and the cells in the control group were cultured in serum-free DMEM under normal conditions during the entire experiment. The MTT assay was used to assess cell viability. The cell morphology was observed by an inverted microscope (Olympus BX-51, Tokyo, Japan).

### Measurement of Intracellular ROS Level

AML-12 cells were plated in 6-well culture plates at a density of 8 × 10^4^ cells/ml and treated with dioscin at concentrations of 50, 100, and 200 ng/ml for 12 h before treatment with Dox. The cells were harvested and then re-suspended in 500 ml of DCFH-DA (10 mM) into each well for 20 min at 37°C. After that, the cells were washed three times with serum-free DMEM, and the images were captured by fluorescent microscopy (Olympus, Japan) with 200× magnification.

### Dox-Induced Hepatotoxicity *In Vivo*

Male C57BL/6J mice weighing 18–22 g were obtained from the Experimental Animal Center of Dalian Medical University (Dalian, China) (SCXK: 2013-0003). All animal experiments were performed according to the guidelines of the Institutional Animal Ethical Committee. Animals were housed in a room under the conditions of constant temperature (22 ± 3°C) and humidity (60%), a 12-h light/dark schedule, and free access to food and water.

After adapting for 1 week, the animals were randomly divided into five groups (*n* = 8): control group, Dox model group, Dio 60 mg/kg + Dox group, Dio 30 mg/kg + Dox group, and Dio 15 mg/kg + Dox group. Dioscin was administered intragastrically at doses of 15, 30, and 60 mg/kg once daily for 14 consecutive days. The mice in the control and model groups were administered with vehicle (0.5% CMC-Na). One hour after dioscin administration on the seventh day, the animals in the model and dioscin-treated groups were injected intraperitoneally with Dox (15 mg/kg) in 0.9% saline (10 ml/kg). After the last dose on the 14th day, the animals were sacrificed, and blood and liver tissues were collected and stored for the test.

### Assessment of Biochemical Parameters

The serum levels of ALS and AST were measured using the commercial kits according to the manufacturer’s instructions. The levels of MDA, SOD, GSH, and GSH-Px in liver tissues were detected according to the manufacturer’s instructions.

### Histological Examination

The liver tissues were fixed in 10% formalin, embedded in paraffin, and sectioned into 5-mm slices. The slices were stained with hematoxylin and eosin (H&E) and photographed by a light microscope (Nikon Eclipse TE2000-U, Nikon, Japan).

### Terminal Deoxynucleotidyl Transferase (TdT)-Mediated dUTP Nick-End Labeling (TUNEL) Assay *In Vivo* and *In Vitro*

Apoptosis detection was executed using the assay kit (Beijing TransGen Biotech Co., Ltd., China) following the manufacturer’s instructions. The cells and tissues were treated with or without dioscin before the fluorescein (green)-labeled dUTP solution was added. Finally, the images were captured by a fluorescent microscope (Olympus, Japan) with 200× magnification and analyzed by the Image J software. The experiment was carried out by the researchers blinded to each group of animals.

### Immunofluorescence Assay

For the immunofluorescence staining of Sirt1, the formalin-fixed and deparaffinized cells and liver tissue sections of mice were incubated with rabbit anti-Sirt1 in a humidified chamber overnight at 4°C. Then, the fluorescein-labeled secondary antibody followed by DAPI (5 μg/ml) was incubated according to the manufacturer’s instructions. TUNEL staining was measured with an *In Situ* Cell Death Detection Kit (TMR Red; Roche, NJ, USA) according to the manufacturer’s instructions. The samples were imaged using fluorescence microscopy (Olympus, Japan).

### Western Blotting Assay

The total protein samples of liver tissues and cells were extracted using cold lysis buffer containing 1 mM of PMSF based on the manufacturer’s protocol, and the protein contents were determined using the BCA Protein Assay Kit. Then the proteins were separated by sodium dodecyl sulfate–polyacrylamide gel electrophoresis (SDS–PAGE) (10–15%), transferred to poly(vinylidene diﬂuoride) (PVDF) membranes (Millipore, USA), blocked, and incubated overnight at 4°C with the primary antibodies listed in **Table 1**. After blocking with 5% dried skim milk for 3 h at room temperature, the membranes were incubated with horseradish peroxidase-conjugated antibody at room temperature for 2 h. Finally, the proteins were detected using an enhanced chemiluminescence (ECL) method and imaged by a BioSpectrum Gel Imaging System (UVP, Upland, CA, USA). Finally, the expression levels of the proteins normalized to GAPDH were detected using the ECL method and ChemiDoc XRS (Bio-Rad, USA).

**Table 1:** The information of the antibodies used in the present work.

Antibody	Source	Dilutions	Company
Sirt1	Rabbit	1:1,000	Proteintech Group, Chicago, USA
FOXO1	Rabbit	1:1,000	Proteintech Group, Chicago, USA
NF-κB	Rabbit	1:1,000	Proteintech Group, Chicago, USA
Keap1	Rabbit	1:1,000	Proteintech Group, Chicago, USA
Nrf2	Rabbit	1:1,000	Proteintech Group, Chicago, USA
HO-1	Rabbit	1:1,000	Proteintech Group, Chicago, USA
P53	Rabbit	1:1,000	Proteintech Group, Chicago, USA
BAX	Rabbit	1:1,000	Proteintech Group, Chicago, USA
BCL-2	Rabbit	1:2,000	Proteintech Group, Chicago, USA
GAPDH	Rabbit	1:2,000	Proteintech Group, Chicago, USA

### Quantitative Real-Time Polymerase Chain Reaction (PCR) Assay

The total RNA samples were extracted from the cells and liver tissues using TRIzol reagent (TaKaRa Biotechnology Co., Ltd., China) according to the manufacturer’s protocol. Then, the purity of the extracted RNA was determined, and cDNA was synthesized using a PrimeScript^®^ RT reagent kit according to the manufacturer’s instructions (TransGen Biotech, Beijing, China). Relative quantitation was performed using the Ct method for recurrent versus primary expression, with GAPDH as an endogenous control, and the fold changes were calculated for each gene. The primers used in the present work are listed in **Table 2**.

**Table 2:** The primer sequences used for real-time PCR assay.

Gene	Forward primer (5′–3′)	Reverse primer (5′–3′)
Human-GAPDH	GAAAGACAACCAGGCCATCAG	TCATGAATGCATCCTTTTTTGC
Human-IL-1β	CCCTGAACTCAACTGTGAAATAGCA	CCCAAGTCAAGGGCTTGGAA
Human-IL-6	ATTGTATGAACAGCGATGATGCAC	CCAGGTAGAAACGGAACTCCAGA
Human-TNF-α	TCAGTTCCATGGCCCAGAC	GTTGTCTTTGAtGATCCATGCCATT
Mouse-GAPDH	TGTGTCCGTCGTGGATCTGA	TTGCTGTTGAAGTCGCAGGAG
Mouse-IL-1β	TCCAGGATGAGGACATGAGCAC	GAACGTCACACACCAGCAGGTTA
Mouse-IL-6	CCACTTCACAAGTCGGAGGCTTA	CCAGTTTGGTAGCATCCATCATTTC
Mouse-TNF-α	TATGGCCCAGACCCTCACA	GGAGTAGACAAGGTACAACCCATC

PCR, polymerase chain reaction.

### Sirt1 siRNA Transfection Experiment *In Vitro*

Transfection experiment was performed on AML-12 cells. The Sirt1 siRNA and control siRNA were dissolved in Opti-MEM and then equilibrated for 5 min at room temperature. The cells were transfected with Sirt1 siRNA or non-binding control siRNA using Lipofectamine 2000 reagent according to the manufacturer’s protocol. Then, the intracellular ROS level, cell apoptosis, and the protein levels of Sirt1, forkhead box protein O1 (FOXO1), kelch-like ECH-associated protein-1 (keap1), NF-E2-related factor 2 (Nrf2), heme oxygenase-1 (HO-1), NF-κB, BCL-2-associated X (BAX), and BCL-2 were measured after 24 h of transfection.

### Statistical Analysis

All data were performed with GraphPad Prism 5.0 software (San Diego, CA, USA) and presented as the mean and standard deviation (SD). Statistically significant differences were determined using one-way analysis of variance (ANOVA) followed by the Newman–Keuls test. Comparisons between two groups were performed using an unpaired Student *t*-test. The results were considered to be statistically significant at *p* < 0.05.

## Results

### Dioscin Rehabilitates Dox-Induced Injury in AML-12 Cells and Mice

As shown in **Figure 1B**, the viability of AML-12 cells treated with 5.0 μM of Dox, compared with the control group, for 24 h was decreased at 72.1%, Thus, Dox at the concentration of 5.0 μM was used to treat the cells. As shown in **Figure 1C**, dioscin at the concentrations of 50, 100, and 200 ng/ml significantly protected cells against Dox-induced injury in a dose- and time-dependent manner. Compared with the Dox group, dioscin on 24-h treatment significantly increased the viability of AML-12 cells. Importantly, dioscin at the concentrations of 50, 100, and 200 ng/ml showed no toxicity to the cells. As shown in **Figure 1D**, the morphological changes of the cells including cell shrinkage and structure caused by Dox were all markedly reversed by dioscin. As shown in **Figure 1E**, dioscin significantly reduced the levels of ALT and AST in mice compared with the Dox group. In addition, H&E staining (**Figure 1F**) revealed that the liver in the control group showed normal architecture, and apparent injuries were found in Dox-treated groups, which were all restored by dioscin.

### Dioscin Inhibits Dox-Induced Oxidative Stress

**Figure 2:**
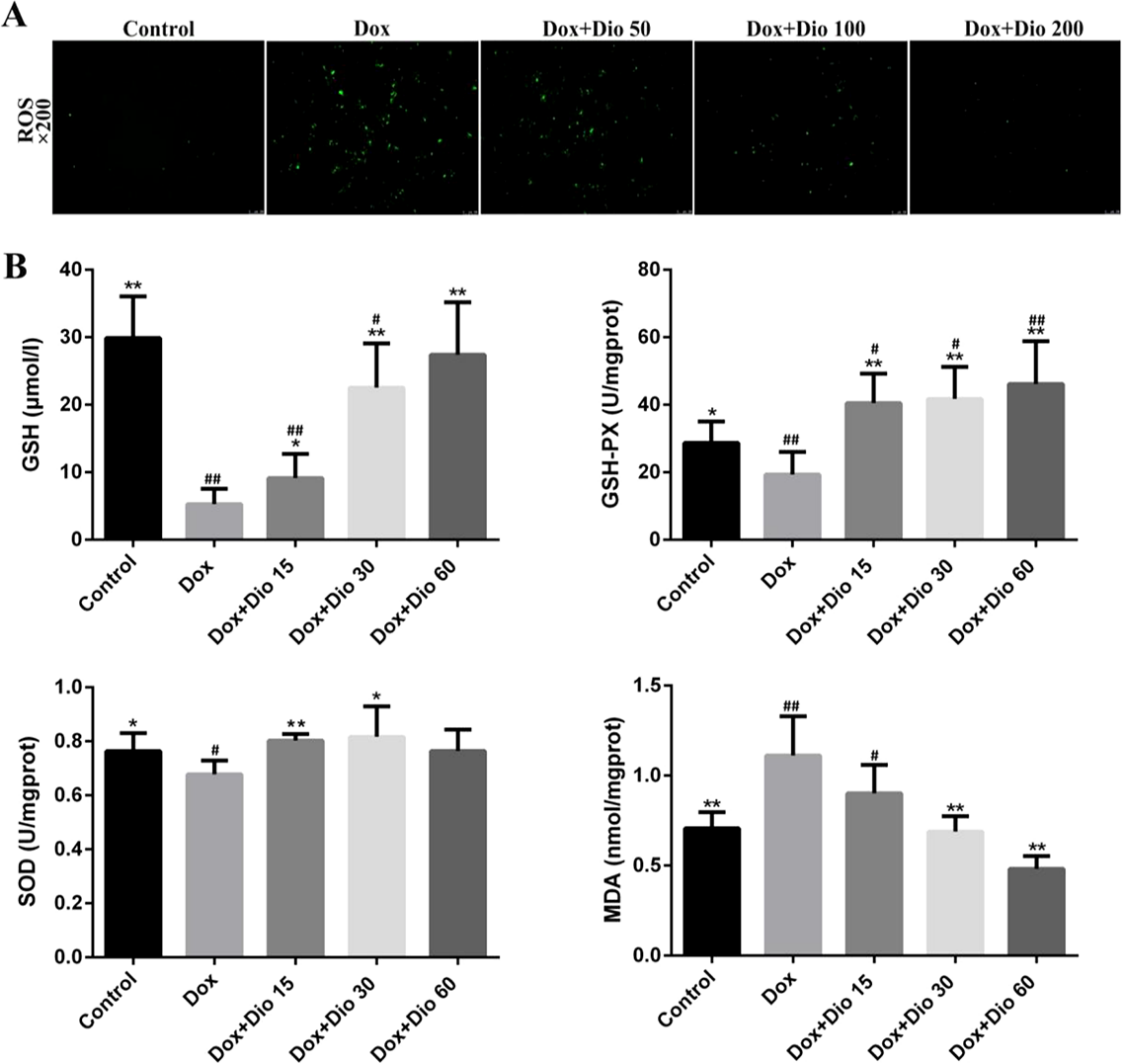
Dioscin attenuates oxidative damage in AML-12 cells and mice. **(A)** Effects of dioscin on intracellular ROS level in AML-12 cells treated with Dox. **(B)** Effects of dioscin on MDA, SOD, GSH, and GSH-Px levels in mice. Data are presented as the mean ± SD (*n* = 3 for *in vitro* test and *n* = 8 for *in vivo* test). *p < 0.05 and **p < 0.01 compared with the Dox group. ^#^ *p* < 0.05, ^##^ *p* < 0.01 compared with the control group. AML-12, alpha mouse liver 12; Dox, doxorubicin; GSH, glutathione; GSH-Px, glutathione peroxidase; MDA, malondialdehyde; ROS, reactive oxygen species; SOD, superoxide dismutase.

Based on immunofluorescence staining shown in **Figure 2A**, after treatment with dioscin at the concentrations of 100 and 200 ng/ml for 12 h, the ROS levels in cells were significantly decreased than those in the Dox group. As shown in **Figure 2B**, dioscin significantly reduced the level of MDA and increased the levels of GSH, GSH-Px, and SOD in mice compared with the model group.

### Dioscin Attenuates Dox-Induced Apoptosis in AML-12 Cells and Mice

**Figure 3:**
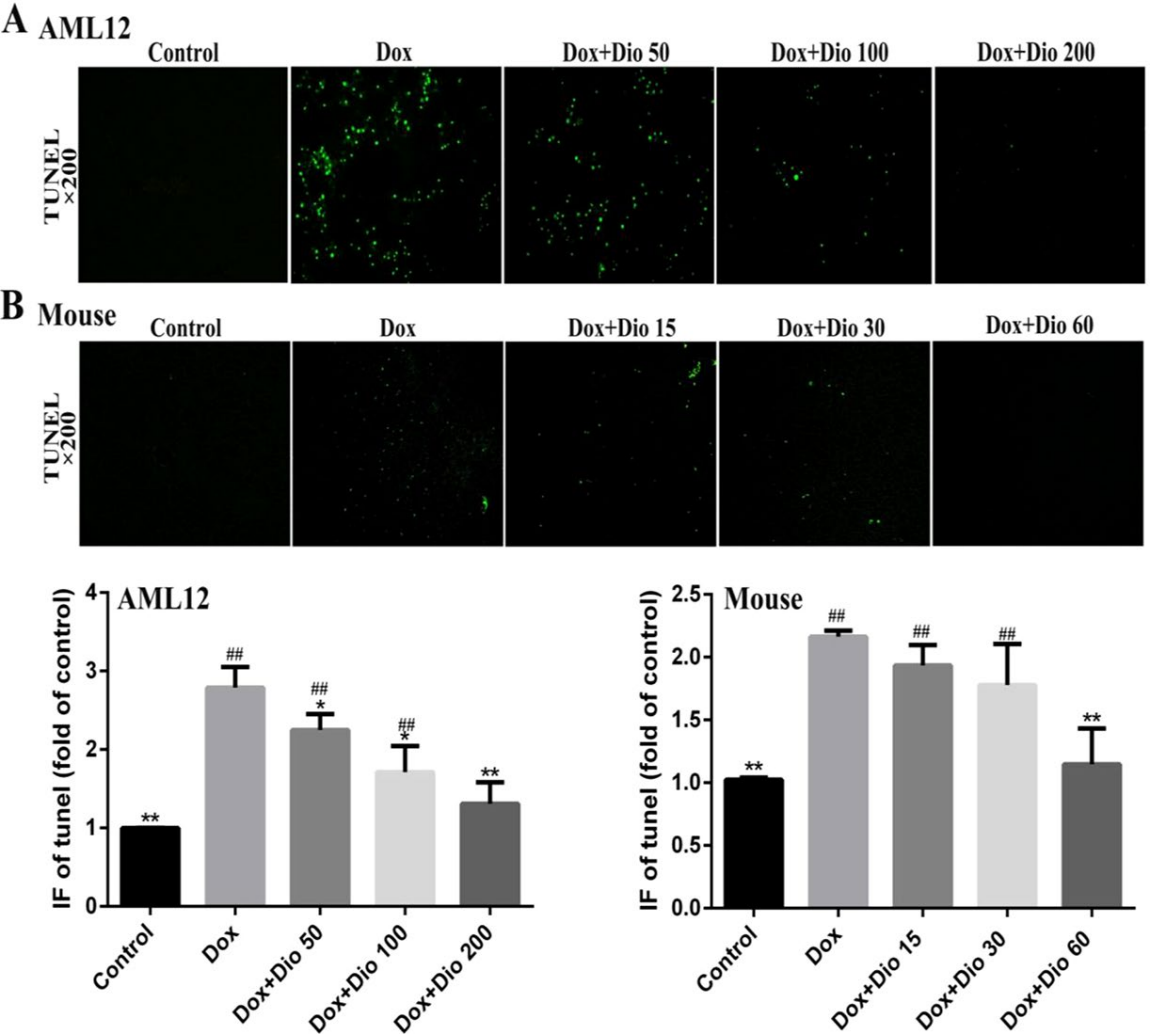
Effects of dioscin on the apoptosis of AML-12 cells and mice using TUNEL assay. **(A)** Dioscin attenuates Dox-induced apoptosis in AML-12 cells. **(B)** Dioscin inhibits Dox-induced liver cell apoptosis in mice. Data are presented as the mean ± SD (*n* = 5 for *in vitro* test and *n* = 8 for *in vivo* test). *p < 0.05 and **p < 0.01 compared with the model group. ^##^ *p* < 0.01 compared with the control group. AML-12, alpha mouse liver 12; Dox, doxorubicin; TUNEL, terminal deoxynucleotidyl transferase-mediated dUTP nick-end labeling.

As shown in **Figures 3A, B**, more TUNEL-positive cells with green fluorescence were observed in Dox-treated groups than in dioscin-treated groups in AML-12 cells and mice.

### Dioscin Increases the Levels of Sirt1 in AML-12 Cells and Mice

**Figure 4:**
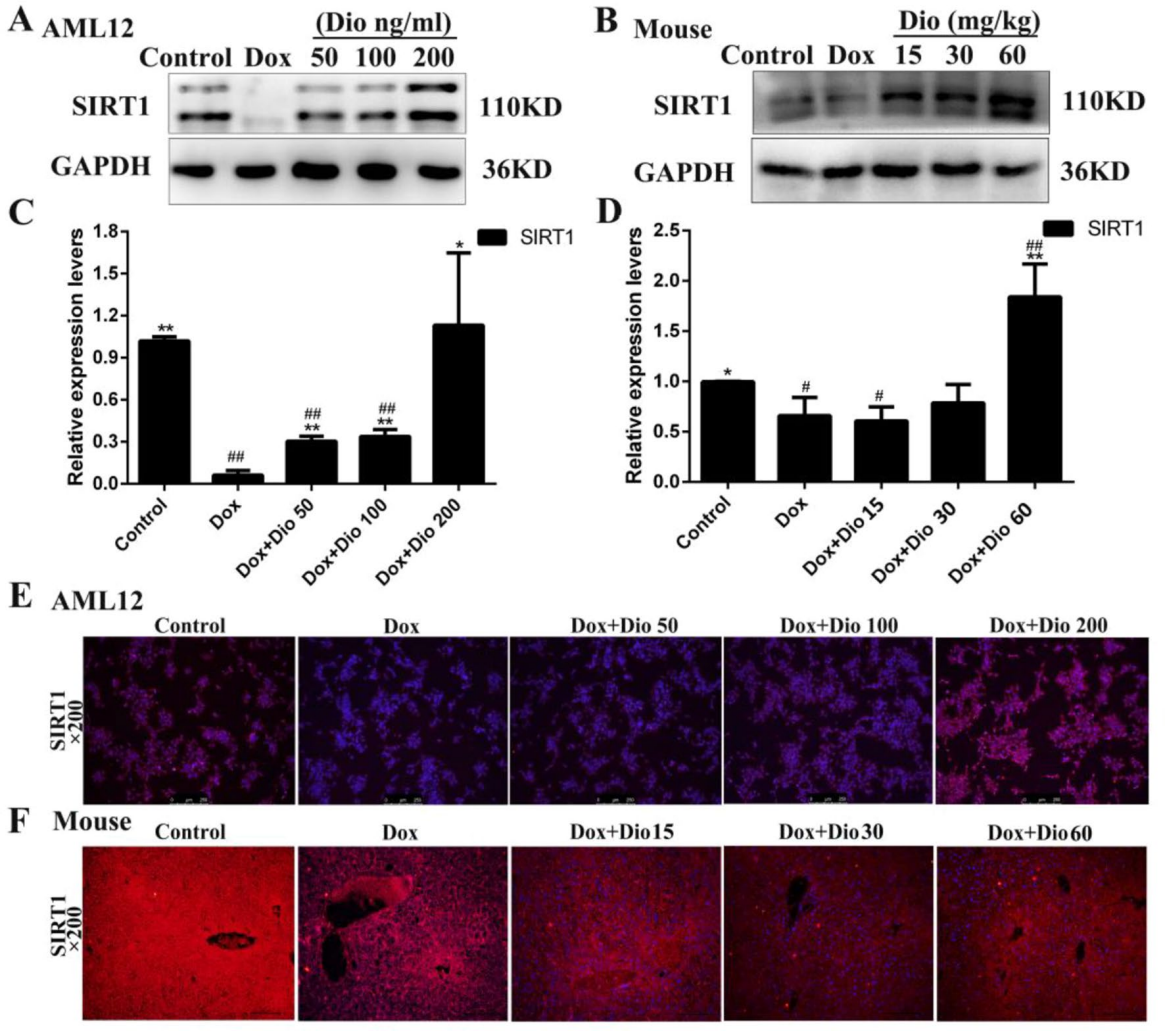
Sirt1 levels are activated in AML-12 cells and mice by dioscin. **(A** *–* **D)** Effects of dioscin on the protein levels of Sirt1 in AML-12 cells and mice. **(E** *–* **F)** Effects of dioscin on the expression levels of Sirt1 based on immunofluorescence staining in AML-12 cells and mice (200× magnification). Data are presented as the mean ± SD (*n* = 3 for *in vitro* test and *n* = 8 for* in vivo* test). *p < 0.05 and **p < 0.01 compared with the model group. ^#^ *p* < 0.05, ^##^ *p* < 0.01 compared with the control group. AML-12, alpha mouse liver 12; Sirt1, silent information regulator 1.

As shown in **Figures 4A, D**, the expression levels of Sirt1 in the model groups were markedly decreased than in the control groups *in vivo* and *in vitro*, which were significantly up-regulated by dioscin based on western blotting assay. In addition, the results of immunofluorescence staining (**Figures 4E, F**) confirmed that dioscin markedly increased the expression levels of Sirt1 in AML-12 cells and mice.

### Dioscin Up-Regulates FOXO1/Keap1/Nrf2 Signal in AML-12 Cells and Mice

**Figure 5:**
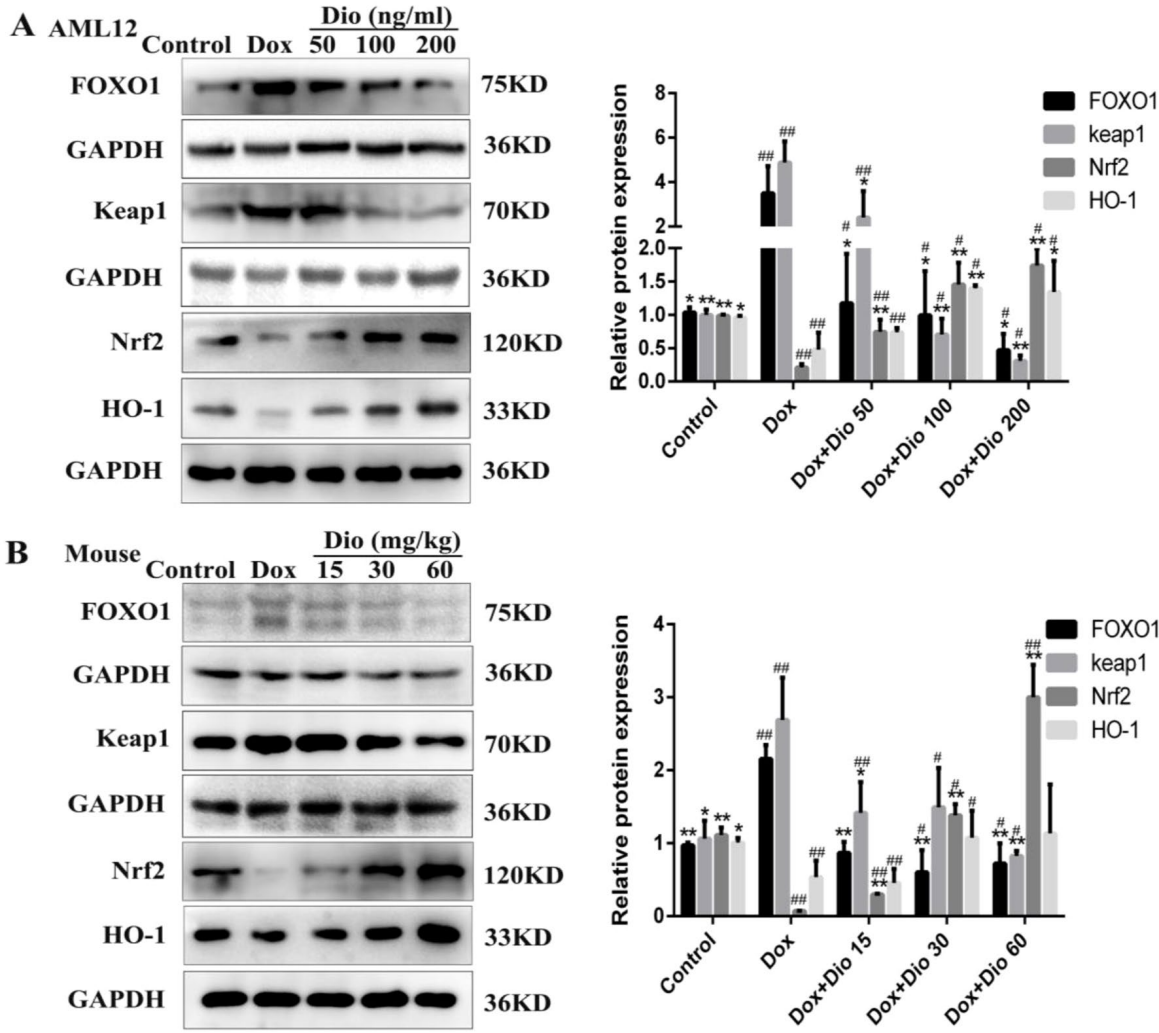
Dioscin suppresses the levels of protein in oxidative stress caused by Dox. **(A)** Effects of dioscin on the protein levels of FOXO1, Keap1, Nrf2, and HO-1 in AML-12 cells. **(B)** Effects of dioscin on the protein levels of FOXO1, Keap1, Nrf2, and HO-1 in mice. Data are presented as the mean ± SD (*n* = 3 for *in vitro* test and *n* = 8 for *in vivo* test). *p < 0.05 and **p < 0.01 compared with the model group. ^#^ *p* < 0.05, ^##^ *p* < 0.01 compared with the control group. AML-12, alpha mouse liver 12; Dox, doxorubicin; FOXO1, forkhead box protein O1; HO-1, heme oxygenase-1; Keap1, kelch-like ECH-associated protein-1; Nrf2, NF-E2-related factor 2.

As shown in **Figures 5A, B**, the protein levels of FOXO1 and Keap1 were significantly decreased, while the protein levels of Nrf2 and HO-1 were increased by dioscin than in the model groups in AML-12 cells and mice.

### Dioscin Inhibits Dox-Induced Inflammation in AML-12 Cells and Mice

**Figure 6:**
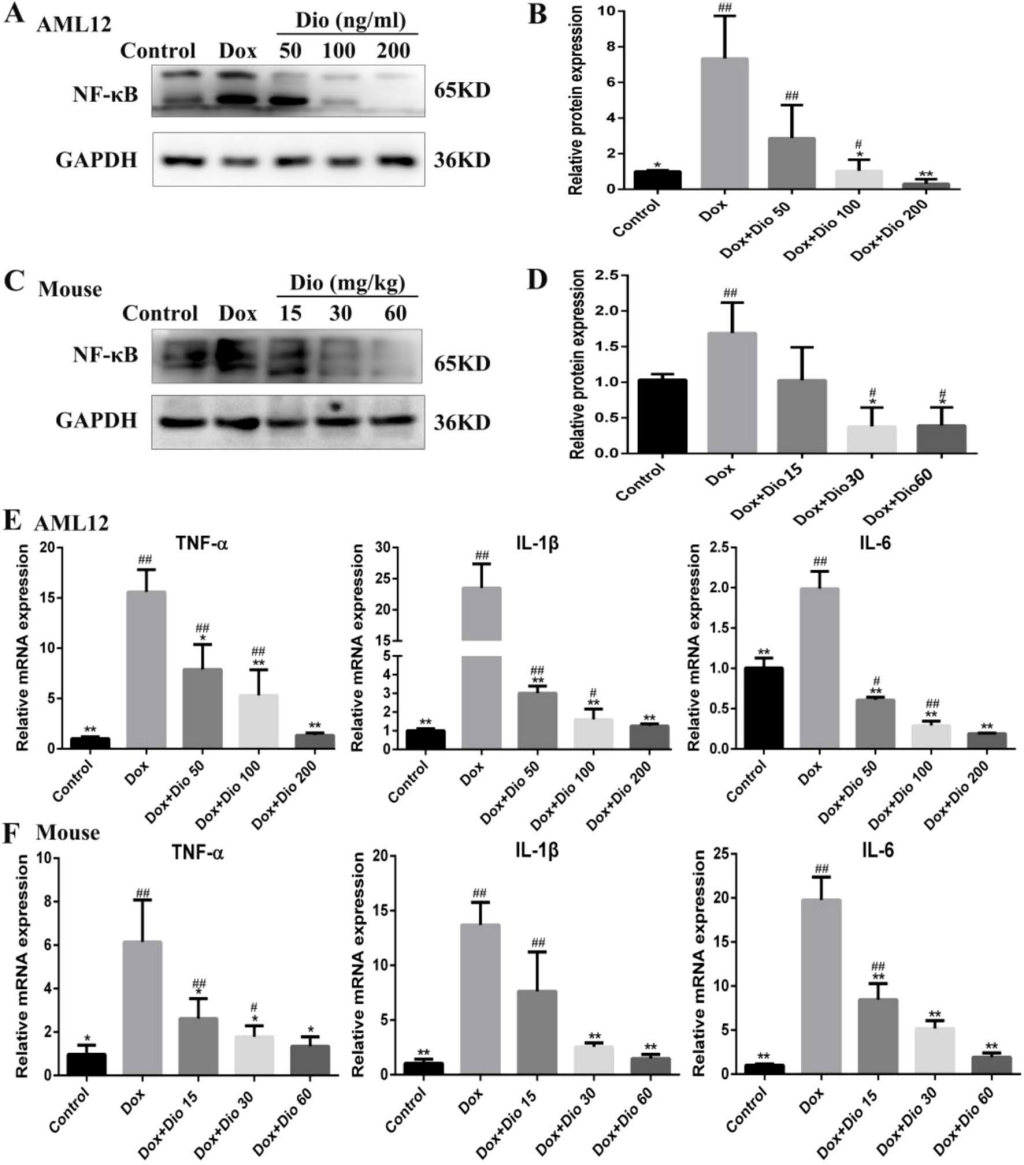
Dioscin ameliorates inflammation *in vitro* and *in vivo*. **(A** *–* **D)** Effects of dioscin on the expression levels of NF-κB in AML-12 and mice. **(E** *–* **F)** Effects of dioscin on the mRNA levels of IL-1β, IL-6, and TNF-α in AML-12 and mice. Data are presented as the mean ± SD (*n* = 3 for *in vitro* test and *n* = 8 for *in vivo* test). *p < 0.05 and **p < 0.01 compared with the model group. ^#^ *p* < 0.05, ^##^ *p* < 0.01 compared with the control group. AML-12, alpha mouse liver 12; IL-1β, interleukin 1β; IL-6, interleukin 6; NF-κB, nuclear factor κB; TNF-α, tumor necrosis factor alpha.

As shown in **Figures 6A–D**, the expression levels of NF-κB were significantly decreased by dioscin in AML-12 cells and mice than in the model groups. As shown in **Figures 6E, F**, the mRNA levels of interleukin 1β (IL-1β), interleukin 6 (IL-6), and tumor necrosis factor alpha (TNF-α) were all significantly decreased by dioscin.

### Dioscin Affects the Protein Levels Related to Apoptosis in AML-12 Cells and Mice

**Figure 7:**
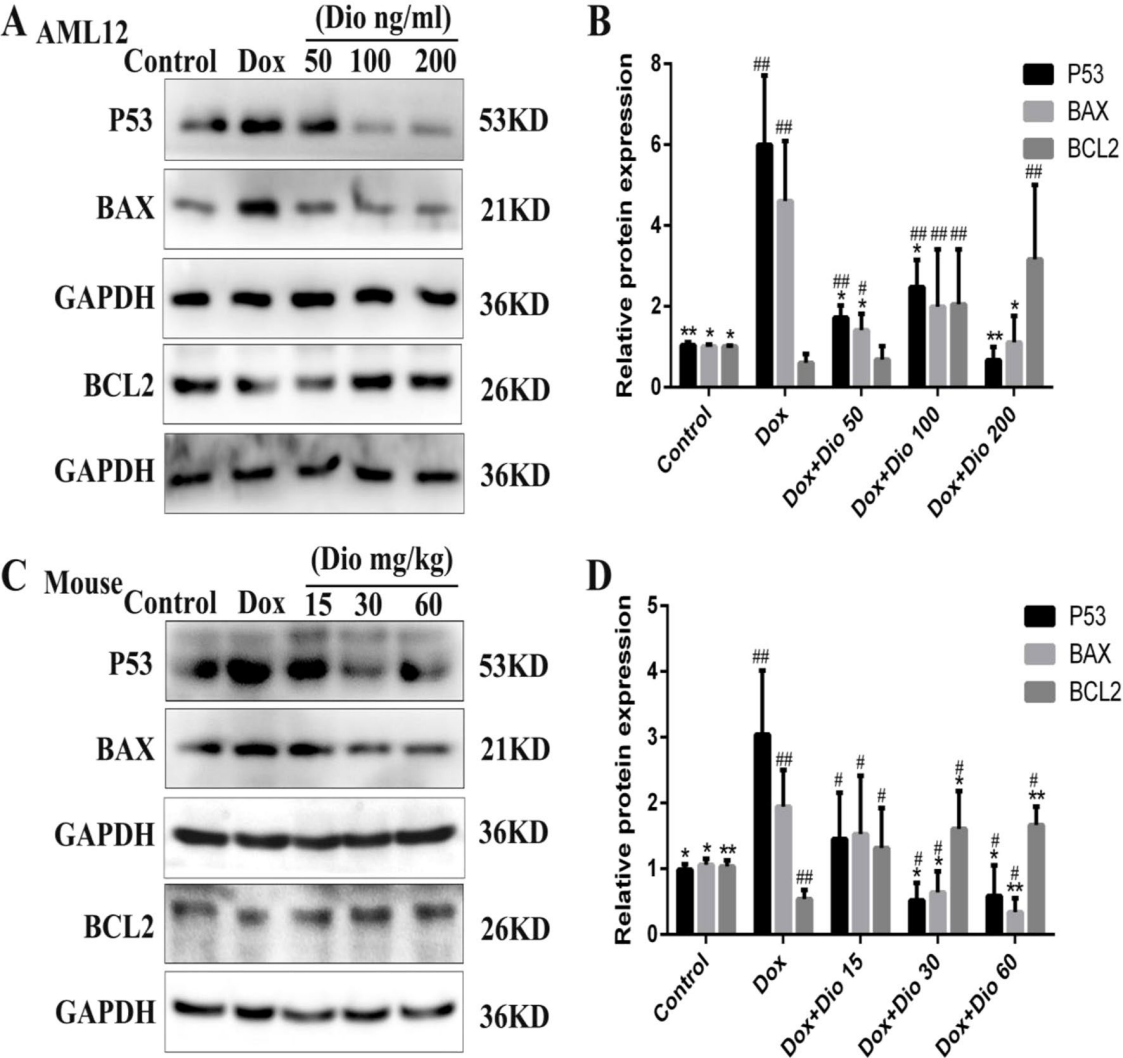
Dioscin regulates P53-mediated apoptosis pathway *in vivo* and *in vitro*. **(A** *–* **B)** Effects of dioscin on the expression levels of BAX and BCL-2 in AML-12 cells. **(C** *–* **D)** Effects of dioscin on the expression levels of P53, BAX, BCL-2 in mice. All data are presented as the mean ± SD (*n* = 3 for *in vitro* test and *n* = 8 for *in vivo* test). *p < 0.05 and **p < 0.01 compared with the model group. ^#^ *p* < 0.05, ^##^ *p* < 0.01 compared with the control group. AML-12, alpha mouse liver 12; BAX, BCL-2-associated X; P53, tumor suppressor P53.

As shown in **Figures 7A–D**, the expression levels of tumor suppressor P53 (P53) and BAX were significantly decreased while the expression levels of BCL-2 were increased by dioscin in a dose-dependent manner than in the model groups.

### Sirt1 siRNA Reverses the Protective Effects of Dioscin on Dox-Induced Cell Injury

**Figure 8:**
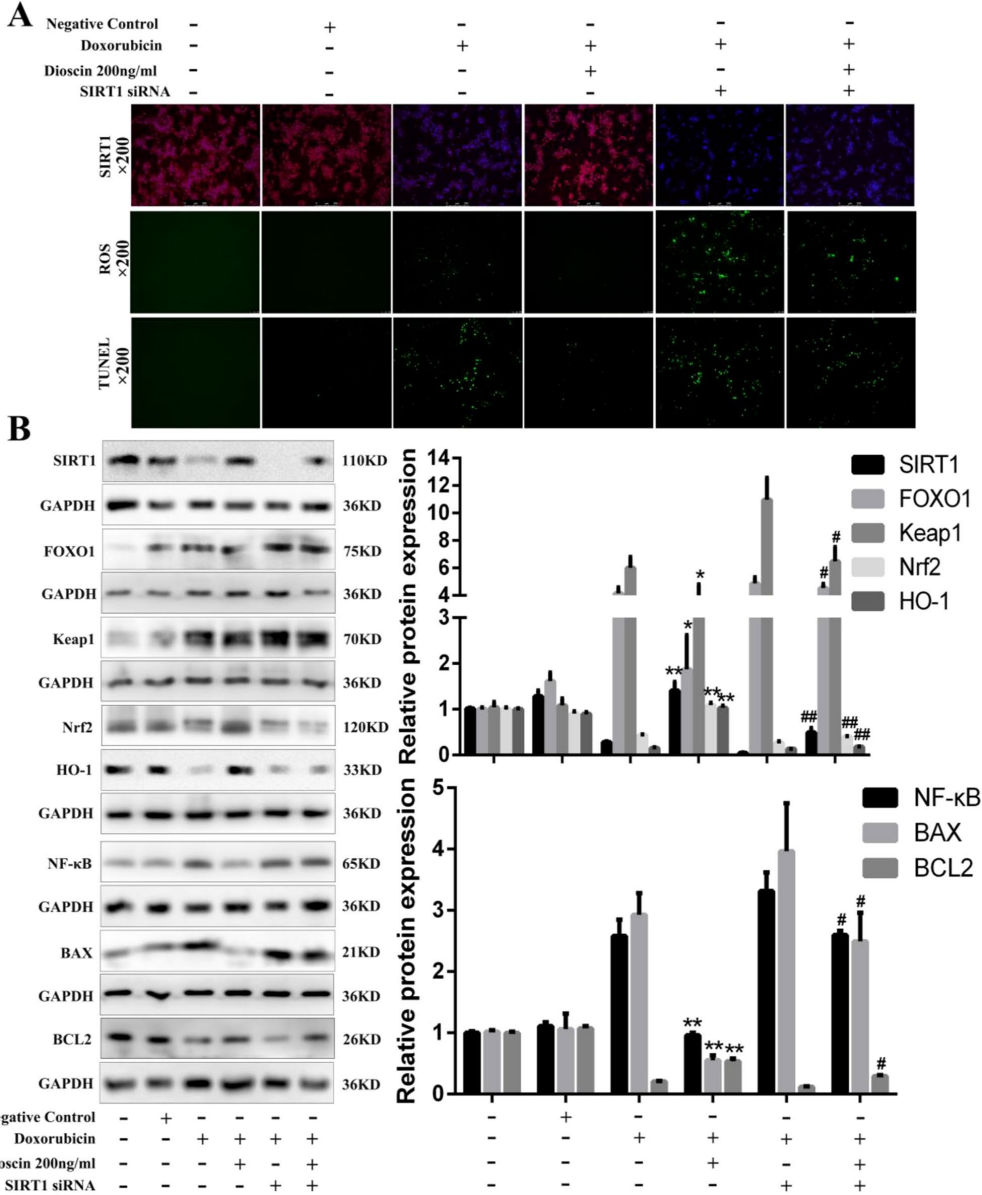
Sirt1 siRNA reverses the effects of dioscin on Dox-induced cell damage. **(A)** Effects of dioscin on Sirt1 expression, ROS level, and cell apoptosis with or without transfection of Sirt1 siRNA *in vitro*. **(B)** Effects of dioscin on the expression levels of Sirt1, FOXO1, Keap1, Nrf2, HO-1, NF-κB, BAX, and BCL-2 after transfection with Sirt1 in AML-12 cells. Data are presented as the mean ± SD (*n* = 5 for *in vitro* test and *n* = 8 for *in vivo* test). ^#^ *p* < 0.05 and ^##^ *p* < 0.01 versus the Dox group; *p < 0.05 and **p < 0.01 versus the Dox group transfected with Sirt1 siRNA. AML-12, alpha mouse liver 12; BAX, BCL-2-associated X; Dox, doxorubicin; FOXO1, forkhead box protein O1; HO-1, heme oxygenase-1; Keap1, kelch-like ECH-associated protein-1; NF-κB, nuclear factor κB; Nrf2, NF-E2-related factor 2; ROS, reactive oxygen species; Sirt1, silent information regulator 1.

To investigate the roles of Sirt1 on the effects of dioscin against Dox-induced liver damage, a transfection approach using Sirt1 siRNA was carried out. As shown in **Figure 8A**, Sirt1 siRNA weakened the dioscin-induced up-regulation of Sirt1 based on immunofluorescence assay and increased the ROS level and the numbers of TUNEL-positive cells. However, dioscin still up-regulated the expression level of Sirt1, inhibited ROS level, and decreased the numbers of TUNEL-positive cells after transfection. Similar results were also found in the expression levels of the proteins related to oxidative stress, inflammation, and apoptosis in Sirt1 signal (**Figure 8B**). These findings confirmed that dioscin suppressed the signaling of oxidative stress, inflammation, and apoptosis *via* up-regulation Sirt1 signal.

## Discussion

Dox, one of the most widely used anticancer drugs, has therapeutic effects on a wide assortment of tumors (Rada et al., 2018). However, Dox can cause a range of significant side effects in normal tissues. Hepatotoxicity, a frequent side effect produced by Dox is of particular relevance under situation of preexisting abnormalities on liver function. Several studies have reported the protective effect of antioxidant agents against Dox-induced hepatotoxicity *via* adjustment of inflammation, oxidative stress, and apoptosis (Jeon et al., 2014; Wang et al., 2019). Dioscin, a naturally derived triterpenoid saponin, displayed the activity on fructose-induced renal damage *via* adjustment of Sirt3-mediated oxidative stress, renal fibrosis, lipid metabolism, and inflammation in rats (Qiao et al., 2017). In addition, dioscin inhibited Dox-induced nephrotoxicity by activating farnesoid X receptor (FXR) to suppress inflammation and oxidative stress, which was proved in NRK-52E cells and rats (Qi et al., 2017). In the present paper, dioscin exerted protective effects against Dox-induced damage on AML-12 cells and protected against Dox-induced liver injury in mice *via* suppression of ROS level, decreasing serum levels of ALT and AST and alleviating histopathological changes. These data implied that dioscin had protective effects by inhibiting Dox-induced hepatotoxicity.

The mechanisms responsible for Dox-induced hepatotoxicity are complex. In recent studies, oxidative stress has been considered as one major mechanism of oxidation-induced hepatotoxicity. It is reported that Dox can induce oxidative stress, which is characterized by ROS accumulation and the decrease of antioxidant defense on oxygen imbalance, culminating in attacking and oxidizing DNA and then inducing liver cell apoptosis (Wang et al., 2019). In the present study, Dox-induced hepatotoxicity produced oxidative stress as evidenced by the high ROS level in cells; high levels of GSH, GSH-Px, and SOD; and a low level of MDA in mice, which were ameliorated by dioscin. Herein, suppression of oxidative stress may be one potent strategy of dioscin against Dox-induced hepatotoxicity.

Many studies have shown that Sirt1 has critical roles against hepatotoxicity, which is a key player in pro-inflammatory context and oxidative stress during acetaminophen-mediated hepatotoxicity (Jeon et al., 2014). Sirt1 is also involved in controlling inflammatory responses, which can adjust NF-κB, a key mediator of pro-inflammatory signaling pathway triggered by cytokines. The latest work has confirmed that activating Sirt1/NF-κB pathway can cause the release of pro-inflammatory cytokines in cisplatin-induced nephrotoxicity (Jung et al., 2012). In the present work, dioscin exerted anti-inflammatory capability that resulted from the decreased levels of NF-κB, TNF-α, IL-1β, and IL-6 *via* increase of Sirt1 expression level. Therefore, inhibiting inflammatory response through adjusting Sirt1/NF-κB signal may be one powerful strategy of dioscin to treat Dox-induced hepatotoxicity.

FOXO1, one of the isoforms in the FOXO family, plays critical roles in cell proliferation and differentiation (Giannakou and Partridge, 2004). Activating Sirt1 can promote FOXO1 expression and transcription from cytoplasm to nucleus (Jung et al., 2012). Moreover, recent studies have shown that resveratrol can reduce Dox-induced cardiomyocytes cell apoptosis through Sirt1-mediated P53 deacetylation, suggesting that Sirt1 activation plays a critical role in apoptosis associated with P53 (Xu R. et al., 2019). The results of this study revealed that dioscin significantly decreased the expression levels of FOXO1, Nrf2, HO-1, P53, and BAX and dramatically up-regulated the expression levels of Keap1 and BCL-2. Taken together, the protective effect of dioscin against Dox-induced liver damage might be attributable to its antioxidative and anti-apoptosis properties through increasing Sirt1 level and activating FOXO1 and P53 pathways. In addition, Sirt1-siRNA transfection tests showed that dioscin showed potent effects against Dox-induced hepatotoxicity *via* regulation of Sirt1/FOXO1/NF-κB signal.

**Figure 9:**
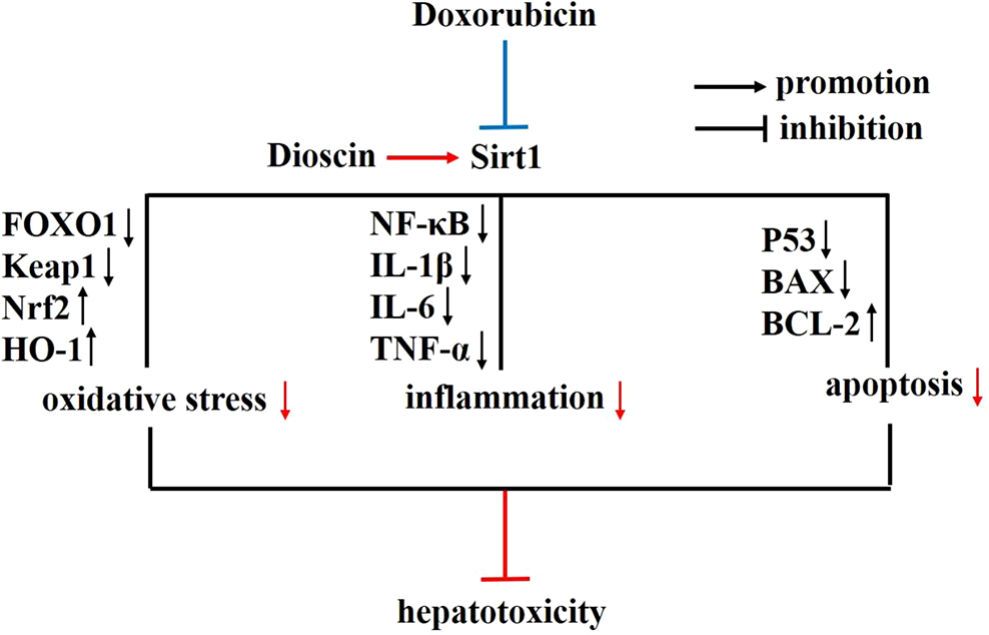
Diagram of the possible mechanism in which dioscin inhibits Dox-induced hepatotoxicity. Dox inhibits the expression of Sirt1, which could trigger cell oxidative stress, inflammation, and apoptosis *via* Sirt1/FOXO1/NF-κB signal pathway to induce hepatotoxicity. However, dioscin promotes the expression of Sirt1 and inhibits the oxidative stress, inflammation, and apoptosis to rescue Dox-induced hepatotoxicity through the Sirt1/FOXO1/NF-κB signal. Blue color indicates the function of Dox, and red color indicates the function and effect of dioscin. Dox, doxorubicin; FOXO1, forkhead box protein O1; NF-κB, nuclear factor κB; Sirt1, silent information regulator 1.

In conclusion, our results indicated that dioscin showed a protective effect against Dox-induced hepatotoxicity through adjusting Sirt1/FOXO1/NF-κB signal (**Figure 9**), which could be developed as a new potential candidate for clinical therapy to treat the disease. However, the deep mechanism of dioscin on Dox-induced hepatotoxicity and its clinical application remains to be further studied

## Data Availability

All datasets generated for this study are included in the manuscript.

## Ethics Statement

The animal study was reviewed and approved by Institutional Animal Ethical Committee. Written informed consent was obtained from the owners for the participation of their animals in this study.

## Author Contributions

SS was responsible for the planning, execution of all experiments, and preparation of the manuscript. LC and HL were responsible for the preparation, isolation, and bioavailability study of dioscin. JC, JL, and ZH were responsible for the immunofluorescence assay, Western blotting, and real-time PCR assays. BZ and XC were responsible for the conceptualization, planning, execution, and troubleshooting of the experiments; preparation of the manuscript; and the financial support.

## Funding

This work was supported by National Natural Science Foundation of China (No. 81700056) and Postdoctoral Science Foundation of China (No. 2018M632878).

## Conflict of Interest Statement

The authors declare that the research was conducted in the absence of any commercial or financial relationships that could be construed as a potential conflict of interest.
